# Cosmeceuticals: The Principles and Practice of Skin Rejuvenation by Nonprescription Topical Therapy

**DOI:** 10.1093/asjof/ojaa038

**Published:** 2020-08-11

**Authors:** Graeme Ewan Glass

**Affiliations:** Associate Professor of Clinical (Plastic) Surgery, Weill Cornell Medical College, New York and Qatar

## Abstract

**Background:**

Aesthetic practice relies on a harmonious relationship between medicine and commerce. Bridging the gap is a large number of skincare products that make therapeutic claims while avoiding the regulatory framework of pharmaceuticals. In this gray area, clinicians find themselves poorly disposed to counsel patients wisely as the industry is expanding faster than empirical evidence of efficacy and safety can be acquired. To serve our patients and engage with industry, we must understand the theoretical principles and evaluate the clinical evidence in practice.

**Objectives:**

The purpose of this paper is to classify cosmeceuticals by method of action, explain how they work in principle with reference to skin aging, and evaluate the clinical evidence for them.

**Methods:**

A literature and cosmetic clinic website search was conducted to establish a list of the most commonly advertised cosmeceuticals, and a peer-reviewed literature search was then conducted to establish the clinical evidence for them.

**Results:**

A huge number of cosmeceuticals are marketed for skin rejuvenation but almost invariably they fall into 1 of 4 categories. These include the induction of tissue repair mechanisms, inflammatory modulation, scavenging of reactive oxygen species, or a combination of the 3. With the exception of retinol derivatives and hydroxy acids, the clinical evidence is limited, despite promising preclinical evidence for several cosmeceuticals.

**Conclusions:**

Cosmeceuticals reside within a highly competitive ecosystem and are often brought to market based on preclinical, not clinical evidence. Success and failure will largely be governed by the establishment of clinical evidence in retrospect.

## THE SKIN REJUVENATION INDUSTRY

The demand for skin rejuvenation strategies has grown exponentially over the last 2 decades with a worldwide annual expenditure projected to reach 10 billion dollars by 2026.^1^ This has fueled the growth in rejuvenation strategies developed along with commercial, as opposed to therapeutic lines. Circumventing the empirical template that is the mainstay of evidence-based medicine sits uncomfortably with most clinicians as ethical practice requires the honest appraisal and communication of benefits and risks. Yet, while this model has blurred the lines between the cosmetic and therapeutic sectors, giving birth to the neologism “cosmeceutical,” it does not follow that there is no therapeutic benefit to be found. Nor does it follow that there is no empirical evidence, despite the knowledge gap. Rather, dermatologist Albert M. Kligman (who coined the term) saw it as a rational necessity on account of the extraordinary rapidity with which new treatments are being developed and an attempt to bridge a gap enshrined in law by the Food, Drug, and Cosmetic Act of 1938^2^ at a time when the distinction was far easier to define. As cosmeceutical skin rejuvenation is ubiquitous in modern grooming, the likelihood is that our patients are following a cosmeceutical skin care regimen. To engage in healthy dialog with our patients and to understand how these cosmeceuticals might influence our surgical outcomes, it is important that we understand what they are and what they do.

## THE PATHOPHYSIOLOGY OF SKIN AGING

All living organs exhibit age-related changes as a result of intrinsic alterations to cell function with the passage of time and number of cell cycle divisions as well as the cumulative effect of extrinsic stressors.^3^ Skin, like any other organ, is subject to intrinsic and extrinsic aging. Intrinsic replicative senescence is a function of progressive telomere shortening with cell division in the absence of telomerase; the enzyme that restores telomere length. The physiological adaptation of cell senescence is not completely understood but it has been proposed as an adaptation to prevent cell immortalization and tumorigenesis.^4^ Progressive lack of cell replacement causes a diminution in tissue quality. Additionally, radiation and toxins cause oxidative stress and cumulative DNA damage, which impairs immune-mediated skin homeostasis.^5^ These can be thought of as extrinsic (and thus to a certain extent, avoidable) inducers of accelerating cell senescence. A number of genes regulate DNA damage surveillance and the intracellular response to oxidative stress. Thus, intrinsic and extrinsic aging are intertwined.^6^ The manifestations of intrinsic aging at a tissue level include thinning of the epidermis and dermis, flattening of the rete ridges, and a relative reduction in type 1 collagen content.^7^ By contrast, the features of skin aging attributed to extrinsic exposures manifest as rhytids caused by the loss of extracellular matrix (ECM) volume and fragmentation of collagen and anchoring fibrils and elastosis (the accumulation of amorphous, irregularly arranged, dysfunctional elastin fibers) with the loss of tone and elasticity, and dyspigmentation (freckles and lentigines) caused by localized changes in melanocyte and melanosome activity.^3,7^ Ultraviolet (UV) exposure also upregulates matrix metalloproteinase (MMP), altering the ratio of MMPs to their inhibitors (tissue inhibitor of metalloproteinase) with progressive loss of dermal collagen.^8^ Together, the histologic, morphologic, and functional features of skin aging have been termed dermatoporosis.^9^

## THE PHYSIOLOGY OF SKIN REPAIR

The response of the skin to acute injury by trauma, chemicals, heat, or radiation, and to attritional and cumulative extrinsic aging, are variants of the same process coordinated by the cross-talk between cells of the innate and adaptive immune systems and the nonimmune cellular components of the epidermis and dermis.^10-12^ Langerhans cells (LC) are a subpopulation of dendritic cells located in the supra-basal layers of the epidermis (stratum spinosum and stratum granulosum) and constitute up to 8% of epidermal cells.^13^ LC are also found within the dermis while migrating to lymphatic channels on their way to presenting antigens to effector (T helper 1 [Th1] and T helper 2 [Th2]) and regulatory T cells. A second population known as inflammatory dendritic epidermal cells are also present in the context of inflammatory skin disorders.^14^

In addition to producing keratin and antimicrobial peptides that form the primary mechanical and microbial barriers, respectively, the keratinocyte is also capable of responding to local damage by the synthesis and release of the proinflammatory cytokines such as tissue necrosis factor (TNF)-α, interleukin (IL)-1β, and IL-6, thereby upregulating the innate immune response in a paracrine manner.^15^ Keratinocytes can also be induced to synthesize chemokines implicated in T cell recruitment.^16^ Both epidermal keratinocytes, dermal fibroblasts and resident cells of innate immunity (LCs, dendritic cells, and mast cells), express surface pattern-recognition receptors (PRRs), including toll-like receptors (TLRs), which identify and bind pathogen-associated molecular patterns (PAMPs) and damage-associated molecular patterns (DAMPs). Such molecules include (lipo) polysaccharides, bacterial lipoproteins, yeasts, and fragments of bacterial DNA (PAMPs) and cell proteins and protein fragments (DAMPs). The consequent intracellular signaling pathways result in cytokine and chemokine expression which, in turn, recruits cells for tissue repair, upregulates the innate immune system, and enhances the presentation of antigenic material to T cells (effector and regulatory) for the deployment of a coordinated adaptive immune response.^17^ The interplay between LCs, dermal dendritic cells, T cells, and keratinocytes is a complex one and adaptable under conditions of variable environmental and psychological stress. For example, it has been established in a murine model that chronic psychological stress results in the upregulation of antigen-presenting cells over time but accompanied by disordered keratinocyte turnover.^18^ This has important implications for the role of psychological well-being as a factor in skin health.

**Figure 1.**
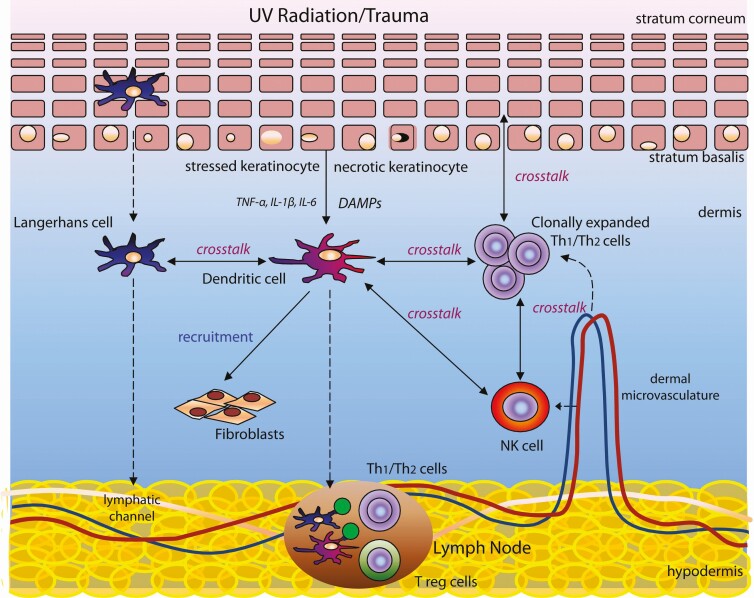
A simplified summary of the immune mechanisms involved in the repair and rejuvenation of photoaged skin. Cells of the innate and adaptive immune systems are recruited by keratinocyte injury as shown. Immune-mediated tissue repair is finely regulated by a high degree of crosstalk between cells involved in protection against further tissue injury and cells involved in implementing tissue repair. DAMPs, damage-associated molecular patterns; NK cells, natural killer cells have features of both innate and adaptive immunity; Th1/Th2, T helper 1 and T helper 2 cells involved in coordinating an adaptive immune response following antigen presentation; T reg, regulatory T cells involved in the maintenance of immune homeostasis, modulating the adaptive immune response and preventing auto-immunity.

**Figure 2.**
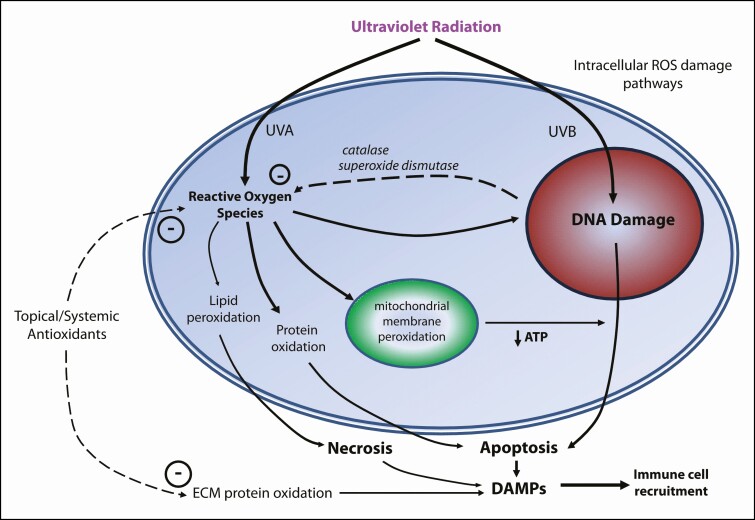
A simplified summary of the mechanism of action of topical and systemic antioxidants in preventing cellular and extracellular damage and inducing the immune response. DAMPs, damage-associated molecular patterns; DNA, deoxyribonucleic acid; ROS, reactive oxygen species; UVA/B, ultraviolet-A (320-380 nanometers and 280-320 nanometers, respectively). The dashed line represents the inhibitory association or negative feedback.

In response to the binding of antigen-presenting cells to Th_1_ cells, the Th_1_ cell initiates a coordinated response to effect tissue repair. Skin-homing effector T cells express cutaneous lymphocyte antigen that binds to the transmembrane migration protein E-selectin, thereby enabling activated T cells to be recruited to the site of tissue injury or pathogenic exposure. The presence of these T cells within the dermis is thought to be associated with a more focused tissue repair response.^10^ However, much work remains to be done to delineate the role of effector T cells in tissue rejuvenation with greater clarity. Figure 1 summarizes immune cell activity in the skin, while Figure 2 summarizes the intracellular and extracellular effects of antioxidants. The mechanism of action of most cosmeceuticals can be understood with reference to these diagrams.^19^

## METHODS

### Search Strategy

A literature review was conducted to establish a comprehensive list of chemical compounds, applied topically, that are marketed under claims of skin rejuvenation and not considered drugs. Additionally, the websites of US and UK-based aesthetic clinics that offer cosmeceutical products and treatments were perused to seek additional cosmeceuticals products. The search was conducted using Google Scholar (no date limit), Pubmed (no date limit), OVID Medline (January 1980 to June 2020), the Cochrane database of systematic reviews, and the Cochrane controlled trials register (searched June 18, 2020). The strategy is detailed in Supplemental Table 1, available online at www.asjopenforum.com. Once a list was assembled, a further literature search was conducted for each in order to establish the existence (or otherwise) of (1) proof of efficacy in principle and (2) peer-review clinical evidence of efficacy and/or safety. Where a proprietary formulation was identified, evidence for the cosmeceutical ingredients, rather than the formulation itself, was sought. Similarly, where the same active cosmeceutical ingredient was derived from different sources, the active ingredient was evaluated rather than the source. This was particularly important when considering botanical oils.

### Exclusion Criteria

Oral dietary supplements marketed for the purpose of skin, hair, and nail rejuvenation (sometimes known as nutraceuticals) were excluded from this review. Like cosmeceuticals, they span the commercial and therapeutic sectors and merit a review on their own. In the case of a chemical compound that can be applied topically (a cosmeceutical) and taken orally (a nutraceutical), only evidence related to topical use was considered here.

## RESULTS

### Cosmeceuticals That Work Principally by Inducing Tissue Repair Mechanisms

#### Vitamin A Derivatives

Vitamin A is an umbrella term denoting a number of related molecules of which retinol and its derivatives (retinoids) are the most important naturally occurring forms. Retinol is highly reactive and unstable and is oxidized to the active form, *trans*-retinoic acid (tretinoin) in vivo as a 2-step process through the intermediary retinaldehyde (retinal). Retinol, retinaldehyde, retinoic acid, their naturally occurring derivatives (retinyl acetate, propionate, and palmitate), and some synthetic derivatives induce skin rejuvenation in a number of ways. These events include the inhibition of IL-6 and interferon-γ, which are anti-inflammatory and favor the recruitment of Th2 cells of the adaptive immune response.^20^ The significance of this in the context of skin rejuvenation is, as yet, unclear. The downstream physiologic process of skin repair and rejuvenation under the influence of immune modulation by retinols involves the synthesis of nuclear transcription factors that, in turn, signal growth factors and cytokines to induce a state of tissue repair. Within the epidermis, they have been shown to regulate keratinocyte growth and differentiation, compaction of the stratum corneum, and the deposition of highly osmotic glycosaminoglycans, including hyaluronic acid.^21,22^ Together, these features produce tactile smoothness and improved skin texture. Within the dermis, retinols reverse the deleterious intrinsic and extrinsic aging effects on collagen homeostasis^8^ by realigning the balance between collagen synthesis and degradation.^23^ Retinols also influence tyrosinase activity and melanosome transfer by melanocytes and sebum production by sebaceous glands leading to improvements in skin texture, rhytids, and dyschromia, and a reduction in skin oiliness and acne vulgaris.^24^ Retinols also increase angiogenesis in intrinsic and photoaged skin.^25^ Retinol, retinaldehyde, and the ester-derivative retinyls are not classed as drugs and, therefore, available for cosmeceutical use, while tretinoin and the synthetic derivatives are classified as drugs.^26^ This highlights the fact that a clear distinction in law between a drug and a cosmetic is, in practice, more nuanced.

#### Tretinoin

The evidence for the clinical efficacy of retinoids is very strong when evaluated against the quality of evidence presented elsewhere in this review. The first English language account of the use of tretinoin in the management of skin aging comes from the studies by Kligman et al^27^ in the 1980s. The first randomized, double-blinded vehicle-controlled study, by Weiss et al,^21^ demonstrated improvements in photoaged skin of the face and forearms with the use of topical tretinoin for 16 weeks. Subsequently, similar randomized, double-blinded vehicle-controlled studies observed similar clinical and histologic benefits of tretinoin.^28-30^ The obvious benefits of topical tretinoin may be longstanding with a maintenance regime.^29,31^ The typical concentration used was between 0.02% and 0.05%, although higher concentrations of up to 0.25% have been trialed safely and may shorten the time to achieve a satisfactory clinical effect.^32^ The side-effect profile includes irritation and redness. None of the high-quality randomized controlled trials reported keratinocytic or melanocytic atypia. While the general consensus is that use should be avoided in women attempting to conceive, it is important to appreciate that studies have not established a causal link between fetal malformation and tretinoin exposure in the first trimester.^33^

#### Retinol, Retinaldehyde, and the Ester Derivatives

Trial data also support the use of the retinol^34-36^ and retinaldehyde^37-39^ in the management of photoaging. While the evidence for efficacy is less voluminous and compelling than for tretinoin that probably reflects the fact that tretinoin is the active form, the precursors appear to be well tolerated.^38,39^ The evidence for the clinical efficacy of the ester derivatives such as retinyl acetate and retinyl palmitate is equivocal.^40,41^

### Synthetic Derivatives

Synthetic derivatives of tretinoin include isotretinoin (second generation), adapalene, bexarotene, tazarotene (third generation), and seletinoid G (fourth generation), and they are classified as drugs. Of these, compelling clinical evidence exists for the role of tazarotene in skin rejuvenation. A large, multicenter, double-blinded, vehicle-controlled trial of topical tazarotene at 0.1% for photoaging revealed significant improvements in multiple indices relative to vehicle-only controls.^42^ A large multicenter, randomized, vehicle-controlled trial comparing various concentrations of topical tazarotene with topical 0.05% tretinoin for photoaging revealed that, at an equivalent concentration, tazarotene performed as well as tretinoin and, for some endpoints, the higher concentration (0.1%) performed better.^43,44^ In a later study using 0.1% tazarotene, they confirmed that this treatment was both safe and efficacious in as little as 2 weeks.^45^ To date, tretinoin and tazarotene remain the only 2 retinoids licensed for the treatment of sun-related skin damage, including fine wrinkles, mottled hyperpigmentation, and tactile roughness.^24^ Seletinoid G also exhibits skin rejuvenation potential in vitro^46^ and in vivo^47^ with a favorable side-effect profile but more clinical data are needed. Evidence for the use of oral isotretinoin in skin rejuvenation, a treatment typically reserved for the management of severe and refractory acne vulgaris, exists^48,49^ but in the context of systemic side-effect profile and the availability of alternatives, there is no rationale for using isotretinoin for this purpose. Adapalene, mainly used as a topical treatment for acne vulgaris, was associated with improvements in sun-related skin damage in a trial of 40 women,^50^ but the evidence remains sparse. Bexarotene is licensed for use in cutaneous T cell lymphoma. There is no trial evidence for the use of bexarotene for skin rejuvenation.

#### Bakuchiol

Bakuchiol is a meroterpene phenol found in the plant *Psoralea corylifolia* that has been proposed as an alternative to the retinoids. Even though the molecular structure is dissimilar, in vitro experiments suggest that it induces a pattern of gene expression in a skin cell line that has many similarities with retinol and has thus been proposed as a functional analog of retinol.^51^ Bakuchiol also exhibits antioxidant and antibacterial activity.^52^ A small, single-blinded clinical study accompanying the in vitro investigation reported that topical bakuchiol at a concentration of 0.5% applied twice daily resulted in improvements in skin aging parameters at 4, 8, and 12 weeks. There was no control arm and the study was performed by Syntheon Ltd, a company specializing in bakuchiol-based cosmeceuticals.^51^ Independent clinical trial data are needed before any conclusions may be drawn about the suitability of bakuchiol in skin rejuvenation.

### Growth Factors

A number of proprietary cosmeceuticals contain combinations of growth factors, including vascular endothelial growth factor, endothelial growth factor, granulocyte, macrophage colony-stimulating factor, and transforming growth factor-beta 1 (TGF-β1), as well as cytokines, including TNF-α, IL-1β, and IL-6. The physiologic rationale for this (either alone or as adjuncts to resurfacing procedures) is well established and described in detail above. The problem with the use of topical growth factors is the poor penetrance of such large molecules through the epidermis.^53^ Nevertheless, some preliminary clinical studies support a role in skin rejuvenation.^54,55^ More evidence is needed.

#### Matrikine Peptides

One of the most important recent advances in matrix biology is the development of our understanding of the role of peptides in matrix synthesis and turnover. Cleavage products of ECM proteins exhibit the potential to induce further protein synthesis, and this phenomenon can be exploited for skin rejuvenation.^56^ GHK (glycine–histidine–lysine tripeptide) is sometimes marketed as “copper peptide” and is a matrikine tripeptide that is active when bound to copper ions. KTTKS (Lysine–Threonine–Threonine–Lysine–Serine) is a pentapeptide marketed under the proprietary name Matrixyl(R), Le Perray-en-Yvelines, France. GEKG (glycine–glutamate–lysine–glycine) is a tetrapeptide. These matrikine peptides have been shown to induce collagen and ECM protein synthesis^57^ and exhibit benefits over growth factors and cytokines as the small molecule size helps them to penetrate the skin.^56^ Not all cosmeceutical peptides induce collagen synthesis. For example, acetyl hexapeptide-8 (Argireline) is actually a fragment of botulinum toxin marketed as an anti-rhytid therapy based on facial muscle relaxation.

In cosmeceutical use, the fatty acid palmitoyl is often chemically bound to the peptide to aid cutaneous absorption by improving lipid solubility. It is also customary to find proprietary formulations containing combinations of matrikines with additional cosmeceutical products, an exhaustive list of which lies out with the scope of this paper. The clinical evidence for the use of matrikine peptides for skin rejuvenation has been reviewed by Gorouhi and Maibach.^57^ In brief, high-quality clinical trial data are lacking.

#### Hydroxy Acids

Traditionally used to produce a controlled ablative tissue injury and thereby the tissue repair cascade,^58^ hydroxy acids are well established topical therapies used to improve rhytids, dyschromia, acne, superficial scarring, and skin texture.^59,60^ Alpha-hydroxy acids (glycolic, lactic, mandelic, benzylic), beta-hydroxy acids (salicylic, lipohydroxy), and α/β hydroxy acids (malic, citric) are now used in topical skin care applications as, at low concentrations, they promote smoother skin by increasing keratinocyte turnover in addition to promoting dermal collagen, elastin, and ECM synthesis^61,62^ without the ablative loss of the epidermal barrier. Polyhydroxy acids such as lactobionic acid and gluconolactone are an additional class of hydroxy acids with a larger molecular structure and slower cutaneous absorption. There is some evidence to suggest that, in consequence, they are less irritant to the skin. 

The evidence for the use of topical hydroxy acids in skin rejuvenation is of relatively high quality. A double-blind, vehicle-controlled, randomized trial investigating the use of 8% glycolic acid and 8% lactic acid creams for photo-damaged skin revealed modest improvements after in some parameters at 22 weeks.^63^ A double-blind, vehicle-controlled, randomized trial using 5% glycolic acid yielded similar results.^64^ The efficacy of α-hydroxy acids in skin rejuvenation has also been reported when combined with other cosmeceutical ingredients.^65^

### Cosmeceuticals That Work Principally by Immune Response Modulation

#### B-Vitamins

Vitamin B3 is an umbrella term denoting a number of related molecules, including nicotinic acid (niacin) and nicotinamide (niacinamide). These molecules are important components of the enzymes nicotinamide adenine dinucleotide (NAD) and nicotinamide adenine dinucleotide phosphate that play critical roles in mitochondrial oxidative phosphorylation. Vitamin B6 and folic acid also synthesize NAD from tryptophan. It has long been established that nicotinamide exerts an anti-inflammatory effect. This has been used to demonstrate the efficacy of topical nicotinamide in the treatment of inflammatory acne vulgaris.^66^ Experimental evidence confirms that nicotinamide and some derivatives selectively inhibit proinflammatory cytokines, including TNF-α, IL-1β, IL-6, IL-12, prostaglandin E2, and nitric oxide (NO), thereby modulating the immune response.^67,68^

Vitamin B derivatives have also been investigated for the management of hyperpigmentation. Nicotinamide has been shown to reduce melanosome transfer in an in vitro keratinocyte/melanocyte coculture model, and these findings were supported by a clinical arm of the same study that reported the efficacy of topical nicotinamide in reducing hyperpigmentation and increased skin lightness when used topically for 4 weeks, relative to the topical vehicle-only control.^69^ Moreover, nicotinamide has also been shown to induce keratinocyte differentiation and regulate lipid synthesis in the stratum spinosum.^70^

#### Beta-glucans

Beta-glucans are a heterogeneous group of glucose polymers (polysaccharides) naturally occurring in a range of bacteria, yeasts, fungi, and plant species, including seaweed and oats. The chemical configuration is based on a β-(1,3)-linked β-d-glucopyranosyl unit with variation provided by β-(1,6)-linked or β-(1,4)-linked side chains of various lengths and configurations.^71^ Beta-glucans are given the term biological response modifiers owing to a range of immunomodulatory activity exhibited by different formulations. Some glucan formulations are PAMPs, triggering the activation and maturation of antigen-presenting cells and stimulating proinflammatory cytokine synthesis and release (especially TNF-α and IL-12) and recruitment of the adaptive immune response by the presentation of antigenic material to the T cells.^72-74^ Recruitment of the adaptive immune system probably reflects an evolutionary adaption enabling rapid recognition and response to fungi and yeasts with mobilization of invariant natural killer T cells.^75^

PRRs for β-glucan PAMPs include Dectin-1, TLRs, complement receptor 3 (CR3), lactosylceramide, and scavenger receptors. Dectin-1 specifically recognizes the β-(1, 3) (1, 6)-glucan pattern, and ligand binding produces a cascade that upregulates the innate immune response. Some breakdown products of larger β-glucan molecules bind CR3 priming the complement cascade.^76^ Intracellular signaling through NF-ƙB is a signaling pathway for the activation of the innate immune response common to ligand binding of both Dectin-1 and TLRs. The solubility of glucans vary, with evidence that soluble β-glucans are more immune-stimulatory than insoluble ones. Some β-glucans have demonstrable antitumor activity.^76^ Additionally, intracellular signaling pathways, and hence biological response, vary with the nature of dendritic cell binding owing to the chemical formulation.^77^ Sterilization by gamma radiation may alter the chemical structure of the glucose polymer, altering its subsequent immunomodulatory effects.^78^ Beta-glucan formulations have now generated interest not only in modulating innate and adaptive immune responses but also as adjuncts in the treatment of viral, fungal, and bacterial infections, malignancies,^79-81^ and inflammatory skin diseases^82^ as well as in accelerating wound healing.^78,80,83^ The practical value of enhancing the innate immune response by the use of β-glucans has also been established for patients undergoing surgery for trauma.^84^

There is currently much interest in the use of β-glucans for skin rejuvenation. It is theorized that β-glucans can induce epithelialization, angiogenesis, fibroblast maturation, collagen, and ECM deposition as well as enhancing surveillance for DNA-damaged autologous cells, tissue injury, and pathogenic invasion by the activation of LCs and dermal dendritic cells. Moreover, keratinocytes have also been shown to express Dectin-1 that, when bound to β-glucan ligands, induces cell migration and proliferation.^85^ Beta-glucans have also been shown to dose-dependently enhance migration and proliferation of human dermal fibroblasts in vitro.^72^ Preclinical studies have provided evidence of enhanced wound healing by increasing the wound tensile strength, which was attributed to the enhanced collagen biosynthesis.^86^ Small clinical case series have reported good outcomes with the use of β-glucan-impregnated gel or dressings in the management of pediatric partial-thickness burns and hard-to-heal wounds, although these data were non-blinded and observational in nature.^87,88^ To date, high-quality randomized controlled trials are lacking.

The second potential role of β-glucans in skin rejuvenation is the protection against oxidative stress by increased synthesis of the protective enzymes superoxide dismutase and catalase as well as the upregulation of superoxide, hydroxyl, peroxide, and peroxynitrite scavenging activity.^83^ Topical β-glucan formulations are also reported to be soothing and, with a large osmotic gradient, help to hydrate the epidermis, improving skin texture and appearance.^89^ The challenge for researchers is to better define the biological response by molecular pattern (most studied β-glucans are impure formulations of a number of polysaccharides) in order to tailor the formulation to optimize the skin rejuvenation potential of this molecular group.^77,90^

### Cosmeceuticals That Work Principally by Scavenging of Reactive Oxygen Species

#### Vitamin C

l-Ascorbic acid (vitamin C) is a potent water-soluble nonenzymatic antioxidant, providing protection against oxidative stress.^91^ Importantly, ascorbic acid also supports functions of both innate and adaptive immunity, enhancing the phagocytic activity of antigen-presenting cells and, as a cofactor for gene regulatory enzymes, influencing proliferation and maturation of T cells and B cells.^92^ Ascorbic acid has also been shown to support the proliferation of keratinocytes and fibroblasts,^92,93^ migration of fibroblasts,^93^ and gene expression of collagen by fibroblasts.^94^ It is also crucially important for collagen cross-linkage and thus tissue strength and stability. It has been hypothesized, although not proven conclusively, that topical application (as opposed to systemic ingestion) can elevate local concentrations in the epidermis and dermis and thus enhance the known beneficial effects on the skin. By enhancing the innate immune response and thus the drive for tissue repair, inducing keratinocyte and fibroblast proliferation, augmenting collagen synthesis and cross-linkage, and protecting against oxidative stress, it is proposed that topical vitamin C can be used as therapy for skin rejuvenation and protection. While there is a theoretically sound basis for this, and while some studies have concluded that the topical vitamin C preparations can protect^95^ and rejuvenate skin,^91^ high-quality, well-designed clinical trials are needed. Finally, the enzyme tyrosinase responsible for the conversion of tyrosine to melanin contains copper molecules. Chemical reactions between ascorbic acid and copper and the antioxidant effect of ascorbic acid on the oxidative reactions in the melanogenic cascade reduce melanin synthesis.^96^ Thus vitamin C has been investigated, either alone or in combination with hydroquinone as a means to remove pigmentary changes associated with sun exposure, with some success.^97,98^

#### Vitamin E

Like vitamin C, vitamin E (α-, β-, γ-, and δ- tocopherols and their related compounds) are potent nonenzymatic antioxidants. Unlike ascorbic acid and its derivatives, the tocopherols are lipid soluble and thus are membrane antioxidants. Vitamin E derivatives are naturally occurring in sebaceous glands and thus sebum has naturally occurring antioxidant properties. The combination of vitamin C and vitamin E has been shown to offer a greater photo-protective effect than either alone.^95^

#### Green Tea Polyphenols

Polyphenols (catechins) found in some plant species including green tea are scavengers of reactive oxygen species, which can exert effects on the skin through the neutralization of free radicals, the prevention of DNA damage, and the accompanying effects on innate immunity, including the induction of a constitutively anti-inflammatory phenotype and accelerated keratinocyte differentiation and turnover.^99^

Oral administration of green tea polyphenols (GTP) has been shown to prevent ultraviolet-B-induced oxidative damage to human fibroblasts in vitro and oxidative damage and MMP expression in murine skin in vivo.^100^ The use of topical GTP has also been shown to exhibit potential as a preventative measure against photocarcinogenesis.^101^ However, this has yet to translate into compelling clinical evidence for skin rejuvenation. A randomized, double-blinded, placebo-controlled trial of an oral GTP supplement, taken twice daily for 2 years, reported no clinical or histologic evidence of improvement in photoaging.^102^

#### Carotenoids

Carotenoids are found in a large number of plant and animal species. They may be divided into xanthophyllic carotenoids, such as lutein and zeaxanthin, and carotenes, such as α-carotene, β-carotene, and lycopene. Carotenoids are potent antioxidants that may be applied topically or taken orally. Both options, and especially combined use, were shown in a clinical trial to improve skin elasticity and hydration.^103^ They are also used as part of sunscreen formulations.^104^ Overall, clinical evidence of efficacy is sparse.

#### Lipoic Acid

Alpha-lipoic acid (ALA) is another potent scavenger of free radicals. In addition, ALA inhibits the nuclear transcription factor NF-ƙB, responsible for proinflammatory signaling.^105^ ALA also exhibits the potential to induce fibroblasts to synthesize collagen in vitro, using the intracellular signaling pathway used by, but not dependent on, TGF-β1.^106^

Following a provisional therapeutic study suggesting that a 12-week course of topical ALA could visibly reduce facial rhytids,^105^ a randomized, double-blind, placebo-controlled split-face trial of topical ALA for photoaged skin, applied twice daily for 12 weeks, reported significant improvements in facial profilometry, as well as clinical and patient-reported observation.^107^

#### Coenzyme Q10 (Ubiquinone)

Coenzyme Q10 is widely used as an oral antioxidant supplement with some evidence of improvements in cardiovascular function and exercise performance.^108^ In the context of skin rejuvenation, a recent randomized, double-blinded, placebo-controlled trial of oral Q10 supplementation (low dose and high dose) reported improvements in periorbital rhytids and skin smoothness over placebo.^109^ Again, more studies involving larger patient numbers are needed. Idebenone is a synthetic analog of coenzyme Q10.

#### Ergothioneine

Ergothioneine (EGT) is a plant-based antioxidant. Keratinocytes express the l-EGT receptor OCTN1, allowing keratinocytes to internalize this molecule.^110^ This constitutes proof-in-principle of the potential of EGT as a topical antioxidant but no clinical studies yet support this.

#### Gamma-Linoleic Acid

The principal cosmeceutical ingredient of evening primrose oil is gamma-linoleic acid (GLA). GLA has been studied mainly as a treatment for atopic dermatitis with mixed results as reviewed by Cronin and Draelos.^111^ No clinical studies support a wider role for GLA for skin rejuvenation.

#### Selenium

Applied topically as l-selenomethionine, selenium is a constituent of intracellular antioxidant enzymes such as glutathione peroxidase and thioredoxin reductase. Anecdotal reports suggest that topical application improves facial rhytids but randomized controlled trial data are lacking.^112^

### Cosmeceuticals That Work in Multiple Ways

#### Copper and Zinc

The role of copper in skin health is multifactorial. Copper ions in a complex with ascorbate are known to induce collagen cross-linkage,^113^ and these important biochemical bonds are crucial for tissue strength and durability. Secondly, copper ions bind with the GHK. This matrikine complex enhances the synthesis of the ECM. Thirdly, copper ions are involved in many intracellular signaling pathways necessary for cell homeostasis. Fourthly, copper ions play an important role in the mitochondrial electron transport chain with the synthesis of adenosine triphosphate (ATP).^114^ Fifthly, copper ions exert antibacterial effects, as do zinc, silver, and gold.^115^ Finally, copper and zinc are integral components of the superoxide dismutase enzyme, which converts superoxide free radicals to hydrogen peroxide and molecular oxygen. Therefore, both trace elements play a role in the prevention of oxidative stress. In addition to these benefits, topical zinc preparations are a mechanical barrier to UV light.

### Other Botanicals

#### Flavonoids

Flavonoids are a diverse group of plant-derived polyphenolic compounds, including the subgroups (pro) anthocyanidins, anthoxanthins, and flavanols. They are found in a wide range of foods, including blueberries, black tea, red wine (grape seeds), and chocolate, and in the plant *Ginkgo biloba*, which is often marketed as a supplement. Grape seed extract, grape seed proanthocyanidins, and resveratrol are other names encountered. They exhibit anti-inflammatory, antioxidant, and antimicrobial activity with low toxicity.^116^ Some flavonoids also exhibit anti-melanogenic effects^117^ and are, therefore, of interest as both cosmeceuticals and nutraceuticals. Clinical trials evidence is sparse.^118^

Isoflavonoids are a group of plant-derived phytoestrogens and a subgroup of the flavonoids.^119^ The principle theory behind their use in cosmeceuticals is in preventing age-related dermal thinning due to downstream activation of growth factor expression.^120^ Again, clinical data are limited.

#### Licorice Root Extracts

Glycyrrhizic acid, liquiritin, isoliquiritin, and glabridin are active ingredients extracted from the licorice root. Sometimes marketed simply as licorice root extract, these molecules actually exert a variety of effects. Glycyrrhizic acid exhibits anti-inflammatory, immunomodulatory, and antiviral effects.^121^ It has also been shown to modulate UV light-mediated damage to dermal fibroblasts.^122^ In a small clinical trial, topical preparations were also shown to improve atopic dermatitis symptoms.^123^ Glabridin, liquiritin, and isoliquiritin exhibit anti-melanogenic effects through the inhibition of tyrosinase and dispersal of melanin, and this has been demonstrated in preclinical studies.^124^ High-quality clinical trials remain elusive.

#### Limonene

Limonene is a molecule found in a large number of botanical oils, including lavender, rosemary, tea tree, peppermint, lemongrass, and citrus oils. The oxidized form may cause contact dermatitis so commercial preparations are usually prepared with antioxidants. It is already found in a wide range of cosmetics and cleaning products. As summarized in a recent review by Vieira et al,^125^ there exists preclinical research to support anti-inflammatory, antioxidant, and antinociceptive effects but no high-quality clinical data to support a role for skin rejuvenation.

#### Curcumin

Principally derived from turmeric, curcumin has also been reported to exhibit anti-inflammatory, antioxidant, and immunomodulatory effects and has been studied extensively as an anti-tumorigenic and anti-inflammatory.^126^ It has been also investigated as a means of reversing UV-induced photoaging in a murine model.^127^ Despite promising preclinical findings, there is a lack of clinical trial data to support a role in skin rejuvenation. A summary of the cosmeceuticals classified by mechanism of action is provided in Table 1.

**Table 1.** Cosmeceuticals Classified by Mechanism of Action

Cosmeceutical	Class/mechanism of action	Source	Proposed use	Summary of evidence quality
Inducers of tissue repair				
Vitamin A derivatives	Regulation of keratinocyte and fibroblast turnover, collagen and ECM synthesis, and melanogenesis and sebum production	Plants and animal products, synthetic	Well-established for use in photoaging, acne	High-quality preclinical and clinical evidence of efficacy and safety
Bakuchiol	Meroterpene phenol	*Psoralea corylifolia*	Functional analog of retinol, also antioxidant and antimicrobial	Limited independent evidence
Growth factors	Including: VEGF, EGF, GM-CSF, TGF-β1, TNF-α, IL-6, and IL-1β	Fetal foreskin fibroblasts, recombinant	Many proprietary formulations available for photoaging	Limited to case series
Matrikines	Peptides	Cleaved from proteins	Various and relates to the source protein-collagen, ECM synthesis, and muscle relaxation	Limited to case series
Hydroxy acids	Α, β, and polyhydroxy acids	Plant sugars, milk	Increase keratinocyte turnover, collagen, and ECM synthesis	High-quality studies but evidence of efficacy modest
Immune response modifiers				
Vitamin B derivatives	Nicotinic acid, nicotinamide	Plant and animal sources	Enhances oxidative phosphorylation enzyme synthesis, inhibits proinflammatory cytokines, and melanosome transfer	Evidence focused on pigmentation rather than photoaging
Βeta-glucans	Polysaccharides	Yeast, bacteria, fungi, and oats	Immune response modification inducing wound healing cascades	Good preclinical evidence. Clinical evidence limited to case series
Flavonoids	Anthocyanidins, flavonols, and resveratrol	Plant-derived, eg, cocoa bean, grape seed, and *Ginkgo biloba*	Anti-inflammatory, antioxidant, and antimicrobial activities	Limited
Isoflavonoids	Phytoestrogens	Legumes, eg, soybeans	Reverse postmenopausal dermal thinning	Limited
Glycyrrhizic acid	Saponin-like compound (fat and water soluble)	Licorice root	Topical anti-inflammatory and antiviral	Limited
Scavengers of reactive oxygen species				
Vitamin C	Nonenzymatic antioxidant	Fruits and vegetables	Prevention of oxidative stress-related photoaging, anti-melanogenic, and ECM collagen cross-linkage	Limited for skin rejuvenation
Vitamin E	Fat-soluble nonenzymatic antioxidant	Vegetable oils, nuts, and seeds	Prevention of oxidative stress-related photoaging and lipid soluble	Used as a component in topical skin rejuvenation formulae
Green tea polyphenols	Polyphenol antioxidant	Green tea	Prevention of oxidative stress-related photoaging	Limited
Carotenoids	Xanthophyllic carotenoids and carotenes	Wide variety of plants	Prevention of oxidative stress-related photoaging	Limited
Alpha-lipoic acid	Organosulfur compound	Vegetables, eg, broccoli and red meat	Prevention of oxidative stress-related photoaging and inhibition of proinflammatory signaling	Provisional evidence encouraging but more studies needed
Coenzyme Q-10	Ubiquinone	Meat, fish, and whole grains	Prevention of oxidative stress-related photoaging	Provisional evidence encouraging but more studies needed
Ergothioneine	Amino acid	Some fungi and bacteria	Prevention of oxidative stress-related photoaging	Limited
Selenium	l-Selenomethionine	Whole grains, nuts, and soybeans	Component of intracellular antioxidant enzymes	Provisional evidence encouraging but more studies needed
Caffeine	Methylxanthine alkaloid antioxidant	Plant based (coffee and black tea).	Topical antioxidant and neural stimulant. Promotes some cell migration and proliferation	Limited
Other				
Copper	Elementary	Formulated	Multi-purpose, including component of intracellular antioxidant enzymes, collagen cross-linkage, GHK matrikine activation, and antibacterial	Used as a component in topical skin rejuvenation formulae
Zinc	Elementary	Formulated	Component of intracellular antioxidant enzymes, compounds provide barrier protection	Used as a component in topical skin rejuvenation formulae
Liquiritin/isoliquiritin/glabridin	Flavonoids	Licorice root	Anti-melanogenic	Limited
Limonene	Hydrocarbon	Plant oils, including lavender, tea tree, peppermint, and rosemary	Antioxidant and anti-inflammatory	Limited
Curcumin	Curcuminoid	Turmeric (*Curcuma longa*)	Antioxidant, anti-inflammatory, and immune-modulatory	Preclinical evidence encouraging but clinical data sparse

ECM, extracellular matrix; EGF, epidermal growth factor; GHK, glycine–histidine–lysine tripeptide; GM-CSF, granulocyte, macrophage colony-stimulating factor; IL, interleukin; TGF-β1, transforming growth factor-beta 1; TNF, tissue necrosis factor; VEGF, vascular endothelial growth factor.

## DISCUSSION

### Cosmeceuticals and Tissue Repair

It seems counterintuitive that many cosmeceuticals are constitutively anti-inflammatory and yet also induce tissue repair, which requires an inflammatory response. There are 2 possible answers here. While acute tissue injury requires a brief proinflammatory stimulus to recruit the means by which the cellular insult can be managed effectively, constitutive expression of proinflammatory cytokines is associated with tissue damage and autoimmune disease. Moreover, certain regenerative and scar-less tissue responses such as fetal wound healing take place in the absence of a proinflammatory response.^128,129^ Indeed, the anti-inflammatory footprint associated with fetal wound healing exhibits some similarities with the way that the response to photoaging is altered by tretinoin, antioxidants, and immune response modifiers. Thus, topical regenerative skin therapies probably rely on attenuating the proinflammatory tendencies of the photoaging repair process inducing a state that, with regular use, is constitutively anti-inflammatory. This is fundamentally different from injecting platelet-rich plasma, lasers, peels, and dermabrasion, which rely on epidermal resurfacing and/or dermal collagen deposition as a result of controlled tissue injury and associated physiological wound repair mechanisms.^11^ A competent immune system is crucial to both mechanisms, highlighting the ingenuity of the immune response and its multifaceted role in tissue regeneration.

There are a number of limitations to this study. Firstly, not all cosmeceuticals have been included. A complete list is impractical owing to the rate at which new products are being marketed. Additionally, it is common to encounter many minor chemical variations of, essentially, the same compound. Only in the case of retinol derivatives, was there evidence of sufficient quality and quantity to justify examination of multiple chemical variations. Moreover, some chemical compounds present in cosmetics are difficult to classify using our working definition of a cosmeceutical. For example, some of the positive effects on the epidermis including smoothness and hydration are attributed to lipophilic substances, which act as both cosmeceutical solvents and emollients. Thus, the list is not exhaustive but is curated from the mass of clinical and commercial information available on the subject. This was thought to be of more practical use to the working clinician rather than a systematic review.

### Cosmeceuticals and the Food and Drug Administration

The Food, Drug, and Cosmetic Act of 1938 made clear that the classification of a product was established by its intended use and not its ingredients. Thus, of paramount importance to regulators are the claims made by the manufacturer about the purpose of the product and what it can do. In the United States, authority over the regulation of both pharmaceuticals and cosmetic products lies with the Food and Drug Administration (FDA). Thus, the FDA may determine from the labeling and advertising whether a cosmetic product (for which there is no requirement to provide evidence of efficacy and safety) should be reclassified as a drug (for which there is). It is, therefore, vital to the commercial interests of the cosmetics industry that cosmeceutical products remain officially classified as cosmetics and not drugs. That said, clinical evidence of efficacy and safety is a potent endorsement of a product that enhances the commercial value, and which is why clinical trials of these products have largely been funded and/or supported by the manufacturers. This, of course, creates its own problems. In Europe, the regulatory framework is more demanding with proof of efficacy and safety required for any marketing claims made by the manufacturers of cosmeceuticals.

## CONCLUSIONS

The modern aesthetic practice blends medicine with commerce, ethically and harmoniously. Consumer demand for skin care products is a poor fit for the pharmaceutical template of product development. Cosmeceuticals are ingredients of skincare products that make therapeutic claims while avoiding the regulatory framework of pharmaceuticals. They reside within a highly competitive ecosystem and are often brought to market without robust clinical evidence. However, the theoretical basis is often secure. Most work either by inducing tissue repair, modulating constitutive immune surveillance, or ameliorating oxidative stress thereby optimizing mitochondrial ATP production. The physiological final common pathway for cosmeceutical products is the maintenance of a constitutively anti-inflammatory skin phenotype, which counters the causes and consequences of photoaging. Success and failure will largely be governed by the establishment of clinical evidence in retrospect, and whether this takes the form of clinical trials data or consumer feedback remains to be seen.

## Supplementary Material

ojaa038_suppl_Supplementary_Table_1
