# Supplementary material for: Cosmeceuticals: The Principles and Practice of Skin Rejuvenation by Nonprescription Topical Therapy
Source: Aesthet Surg J Open Forum. 2020 Aug 11;2(4):ojaa038. doi: 10.1093/asjof/ojaa038 (PMC9905273; doi:10.1093/asjof/ojaa038)
Supplement: ojaa038_suppl_Supplementary_Table_1 [file ojaa038_suppl_supplementary_table_1.docx]

**Supplemental Table 1**. Search Terms

| 1 | "cosmeceutical" |
| --- | --- |
| 2 | "skin rejuvenation" |
| 3 | "skin enhancement” |
| 4 | "anti-aging" |
| 5 | "anti-photoaging" |
| 6 | "rejuvenation" |
| 7 | "skin repair" |
| 8 | "antioxida*" |
| 9 | 1 OR 2 OR 3 OR 4 OR 5 OR 6 OR 7 OR 8 |
| 10 | topical$ |
| 11 | 9 AND 10 |
| 19 | limit 18 to year="1980* – Current" |

*1980 chosen as the date the term “cosmeceutical” was coined
